# Effect of Pneumococcal Conjugate Vaccines on Viral Respiratory Infections: A Systematic Literature Review

**DOI:** 10.1093/infdis/jiae125

**Published:** 2024-03-11

**Authors:** Ingrid T Sepúlveda-Pachón, Eileen M Dunne, Germaine Hanquet, Marc Baay, Sonia Menon, Luis Jodar, Bradford D Gessner, Christian Theilacker

**Affiliations:** Epidemiology Department, P95 Epidemiology and Pharmacovigilance, Leuven, Belgium; Global Vaccines and Antivirals, Pfizer Inc, Collegeville, Pennsylvania; Epidemiology Department, P95 Epidemiology and Pharmacovigilance, Leuven, Belgium; Epidemiology Department, P95 Epidemiology and Pharmacovigilance, Leuven, Belgium; Epidemiology Department, P95 Epidemiology and Pharmacovigilance, Leuven, Belgium; Global Vaccines and Antivirals, Pfizer Inc, Collegeville, Pennsylvania; Global Vaccines and Antivirals, Pfizer Inc, Collegeville, Pennsylvania; Global Vaccines and Antivirals, Pfizer Inc, Collegeville, Pennsylvania

**Keywords:** lower tract respiratory infection, LRTI, pneumonia, viral, pneumococcal conjugate vaccine

## Abstract

**Background:**

In addition to preventing pneumococcal disease, emerging evidence indicates that pneumococcal conjugate vaccines (PCVs) might indirectly reduce viral respiratory tract infections (RTIs) by affecting pneumococcal-viral interactions.

**Methods:**

We performed a systematic review of interventional and observational studies published during 2000–2022 on vaccine efficacy/adjusted effectiveness (VE) and overall effect of PCV7, PCV9, PCV10, or PCV13 against viral RTIs.

**Results:**

Sixteen of 1671 records identified were included. Thirteen publications described effects of PCVs against viral RTIs in children. VE against influenza ranged between 41% and 86% (n = 4), except for the 2010–2011 influenza season. In a randomized controlled trial, PCV9 displayed efficacy against any viral RTI, human seasonal coronavirus, parainfluenza, and human metapneumovirus. Data in adults were limited (n = 3). PCV13 VE was 4%–25% against viral lower RTI, 32%–35% against coronavirus disease 2019 outcomes, 24%–51% against human seasonal coronavirus, and 13%–36% against influenza A lower RTI, with some 95% confidence intervals spanning zero. No protection was found against adenovirus or rhinovirus in children or adults.

**Conclusions:**

PCVs were associated with protection against some viral RTI, with the strongest evidence for influenza in children. Limited evidence for adults was generally consistent with pediatric data. Restricting public health evaluations to confirmed pneumococcal outcomes may underestimate the full impact of PCVs.

## Teaser

Emerging evidence indicates that pneumococcal conjugate vaccines (PCVs) not only reduce pneumococcal disease burden but also might indirectly reduce viral respiratory tract infections by affecting pneumococcal–viral interactions. We summarized available evidence on PCV protection against virus-related respiratory tract infections in children and adults.

Respiratory tract infections (RTIs) represent a key global health concern as they cause substantial morbidity and mortality worldwide, predominantly in children under 5 and older adults [1]. They are typically categorized as upper respiratory tract infections (URTIs), which are usually milder but more frequent, or lower respiratory tract infections (LRTIs), which include pneumonia, bronchiolitis in children, and bronchitis. Community-acquired URTI and LRTI may be caused by a wide range of bacterial, viral, or fungal pathogens but are commonly caused by viruses, including influenza, respiratory syncytial virus (RSV), coronaviruses, human metapneumovirus (hMPV), parainfluenza virus, enterovirus, and adenovirus [2].

Bacterial and viral coinfections are common in the respiratory tract [3, 4] and have been associated with increased severity [5]. Historically, viral infections have been considered to precipitate secondary bacterial, and particularly pneumococcal, LRTIs through various mechanisms [6]. The classic example of this secondary bacterial infection was the association between influenza and secondary pneumonia cases caused by bacterial pathogens, including pneumococci, during the 1918–1919 influenza pandemic [7]. More recent evidence indicates that interactions between bacteria and viruses in respiratory infection can be bidirectional [8–10]. Given the synergistic interactions between bacterial and viral pathogens, vaccination against either bacterial or viral pathogens might have broader benefits than reducing etiologically confirmed disease due to the target pathogen.

Pneumococcal conjugate vaccines (PCVs) have been licensed for the prevention of pneumococcal disease caused by the serotypes included in the vaccine formulation (Supplementary Table 1). Randomized clinical trials and observational effectiveness studies have reported that PCVs reduce the incidence of all-cause pneumonia beyond that attributed to serotype-specific pneumococcus [11–13]. One explanation for this underestimation is that PCVs are also reducing viral-associated pneumonia episodes for which vaccine-type pneumococci play a role in the causal chain [11, 14, 15]. We conducted a systematic literature review to summarize the evidence on the effects of PCVs against virus-related RTI in children and adults. We did not cover the effect of the 23-valent polysaccharide vaccine because its lack of impact on carriage may imply different mechanisms for any effect found.

## METHODS

We conducted a systematic literature review, adhering to the Preferred Reporting Items for Systematic Review and Meta-Analyses (PRISMA). The review is registered in the International Prospective Register of Systematic Reviews (PROSPERO; registration number CRD42022339625).

### Search Methodology

Eligibility criteria were developed using the PICOTS (population, intervention, comparator, outcomes, time frame, setting) framework (Supplementary Table 2). Studies were eligible if they reported on any vaccine effect (direct or overall effect) of PCV on viral LRTI in adults and children and on viral URTI in children. Direct effect is the protection provided by the vaccine in vaccinated individuals; it is estimated by comparing the risks or rates of disease in vaccinated and unvaccinated individuals exposed to the same vaccination program, and comprises vaccine efficacy and vaccine effectiveness (VE) [16]. Overall effect is the effect of the vaccination program on the entire population (including vaccinated and unvaccinated persons) and is estimated by comparing the risks or rates of disease in populations with and without a vaccination program [16]. This is also referred to as vaccination impact. Inclusion and exclusion criteria are described in Supplementary Table 2.

Medline and Embase databases were systematically searched for publications from January 2000 to June 2022 in English, Spanish, French, and Portuguese. The detailed search strategy and search terms are presented in Supplementary Table 3. Additional studies were identified through hand searching in references of included publications, systematic literature reviews on LRTI, a grey literature search in Opengrey and Greylit, and a search of conference abstracts of the Respiratory Syncytial Virus Network (ReSViNET), IDWeek, European Congress of Clinical Microbiology and Infectious Diseases (ECCMID), European Society for Paediatric Infectious Diseases (ESPID), and International Society of Pneumonia and Pneumococcal Diseases (ISPPD) from 2020 to June 2022.

### Study Selection and Data Extraction

Retrieved articles were screened independently by 2 reviewers based on title and abstract, and discrepancies were solved by a third reviewer. Full-text screening was performed by one reviewer and a quality check of 10% of the screened articles was done by a second reviewer.

Data extracted included clinical outcome (type of RTI), study design, study period, treatment setting, population description, type of PCV, viral pathogen, type of effect (direct or overall effects), and effect measures (efficacy, risk ratio, etc). All extractions were quality-checked by a senior epidemiologist. Clinical outcomes (type of RTI, such as pneumonia, community-acquired pneumonia [CAP], LRTI, severe acute respiratory illness, bronchiolitis, etc) varied by study and were defined by the original studies.

### Risk of Bias Assessment

Four specific adapted tools were used according to the study design of the included studies: the Risk of Bias 2 Tool by the Cochrane Collaboration for randomized trials, the Newcastle-Ottawa Scale for cohort and case-control studies, and the National Institutes of Health checklist for before-after studies.

### Data Analysis

Data were summarized descriptively and stratified by age group, type of RTI, type of vaccine effect (direct or overall), and pathogen. The adjusted VE was reported unless only the crude VE was provided. When results from both intent-to-treat and per protocol analyses were reported by the original study, results from the intent-to-treat analyses were considered the primary result and included in summary tables. When applicable, VE and the corresponding 95% confidence interval (CI) were calculated using the relative risk (RR), odds ratio (OR), or hazard ratio (HR) reported in the articles as follows: VE =(1 – ratio) × 100%. For the measure of overall effect, the incidence rate ratio (IRR) and the corresponding 95% CI were calculated, when applicable, from the incidence rates provided per period: IRR = post-PCV incidence rate/pre-PCV incidence rate. In one study, it was calculated from the vaccine impact (VI) reported, as follows: IRR = (1 – VI). Due to differences in vaccines, outcomes, study designs, and population of included studies and subsequent data heterogeneity, we did not attempt to perform meta-analyses.

## RESULTS

### Study Description

**Figure 1.**
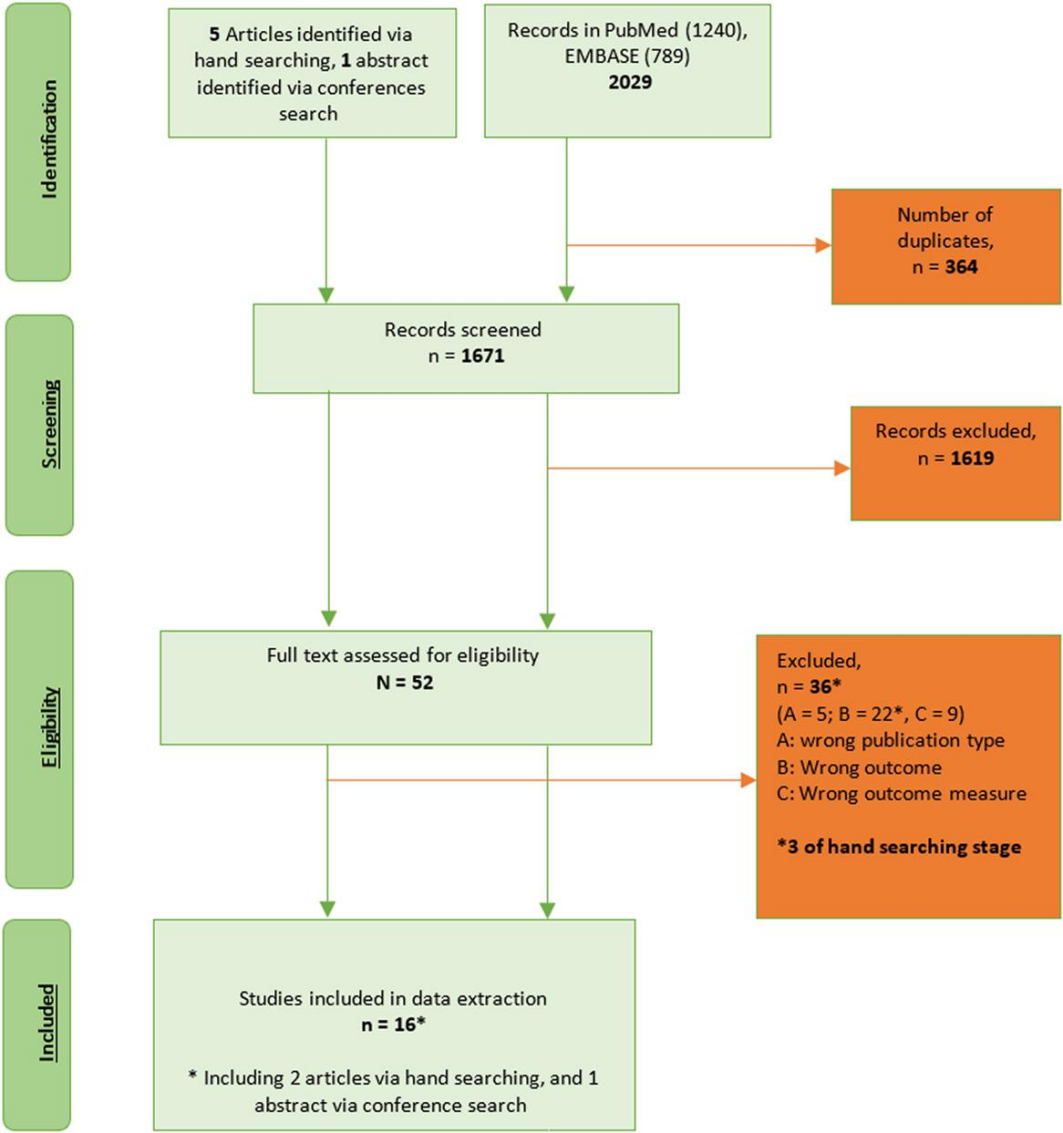
Preferred Reporting Items for Systematic Review and Meta-Analyses (PRISMA) flowchart.

Overall, 1671 manuscripts were identified, of which 16 manuscripts were eligible and contributed to the final analysis (see PRISMA chart in Figure 1, Table 1, and Supplementary Table 4). Thirteen manuscripts were found in children: 7 described direct effect [11, 17–21, 29] and 6 reported overall effect [22–27]. Three publications described direct effects in adults [14, 28, 30]. The geographic distribution was as follows: Africa (n = 4) [11, 17, 19, 21], Asia (n = 1) [27], Europe (n = 5) [18, 20, 26, 28, 29], North America (n = 4) [14, 22, 23, 30], and Oceania (n = 2) [24, 25]. Four studies reported on any RTI [18, 20, 29, 30], and 12 on LRTI, including pneumonia and nonpneumonia LRTI outcomes [11, 14, 17, 19, 21–28] (outcome definitions provided in Supplementary Table 5). Study design included randomized controlled trials (RCTs; n = 5) [11, 17, 18, 21, 28] and observational studies including comparison of pre-PCV and post-PCV introduction incidence (n = 6) [22–27] (including time series analysis [n = 1] [23]), cohort (n = 2) [20, 30], case-control (n = 2) [14, 29], and case-population design (also called screening method [n = 1] [19]). Three manuscripts reported on different viral outcomes for the same RCT investigating the efficacy of PCV9 in South African infants [11, 17, 21].

**Table 1.** Description of Included Studies Investigating the Effects of Pneumococcal Conjugate Vaccines on Virus-Associated Respiratory Infections

Reference	Country	Population	Study Design	PCV	Study Years	Clinical Outcome (RTI Type)	Viral Pathogen	Overall Sample Size	No. of Viral Cases
Children									
Direct effect									
** **Madhi, 2004 [11] ^a,b^	South Africa	28 to 84 d at enrollment	RCT	PCV9	1998–2001	Pneumonia	AdV, influenza, PIV, RSV, and virus unspecified	39 836	627
** **Madhi, 2006 [17]^a,b^	South Africa	28 to 84 d at enrollment	RCT	PCV9	1998–2002	LRTI, bronchiolitis, pneumonia	hMPV	39 836	195
** **Jansen, 2008 [18]^a^	Netherlands	18 to 72 mo	RCT	PCV7^c^	2003–2005	RTI	Influenza	579	48
** **Dominguez, 2013 [29]^d^	Spain	6 mo to 5 y	Case-control	PCV7/10/13	2009–2011	RTI	Influenza	536	194
** **Abadom, 2016 [19]^d^	South Africa	2 d to 5 y	Case-population (screening)	PCV7/13	2009–2012	SARI	Influenza	5 685 452	1229
** **Karppinen, 2019 [20]^d^	Finland	≤2 y	Cohort	PCV10	2008–2012	RTI	HRV and virus unspecified	368	1227
** **Nunes, 2021 [21]^b,d^	South Africa	1 to 23 mo	RCT	PCV9	1998–2006	Pneumonia	HCoV and virus unspecified	39 836	693
** **Overall effect									
** **Foote, 2015 [22]	US	≤4 y	Pre and post	PCV7/13	1998–2010	LRTI	RSV	NR	NR
** **Weinberger, 2015 [23]	US	≤23 mo	Time series analysis	PCV7	1997–2009	LRTI	RSV	NR	729 526
** **Fathima, 2018 [24]	Australia	≤16 y	Pre and post	PCV7/13	2000–2012	Pneumonia	AdV, influenza, hMPV, PIV, PV, RSV	469 589	2166
** **Binks, 2020 [25]	Australia	7 d to 12 mo	Pre and post	PCV7/10/13	2006–2015	Pneumonia	RSV and virus unspecified	14 594	394
** **Sigurdsson, 2020 [26]	Iceland	≤3 y	Pre and post	PCV10	2005–2015	Pneumonia	Influenza and virus unspecified	51 264	122
** **Do, 2022 [27]	Mongolia	≤1 y	Pre and post	PCV13	2015–2020	Pneumonia	Influenza, RSV	5680	2337
Adults									
Direct effect									
** **Huijts, 2018 [28]^a^	Netherlands	≥65 y	RCT	PCV13	2008–2013	CAP	AdV, HBoV, HCoV, influenza, hMPV, PIV, HRV, RSV, virus unspecified, or mixed (2 virus or virus + bacteria)	84 496	221
** **Lewnard, 2022 [30]^d^	US	≥65 y	Cohort	PCV13	2020	RTI	SARS-CoV-2	531 033	3677
** **Lewnard, 2023 [14]^d^	US	≥18 y	Case-control	PCV13	2016–2019	LRTI, pneumonia, and nonpneumonia LRTI	AdV, HCoV, EV, influenza, hMPV, PIV, RSV, virus unspecified	241 743	17 416

Abbreviations: AdV, adenovirus; CAP, community-acquired pneumonia; CPR, case-population ratio; EV, enterovirus; HBoV, human bocavirus; HCoV, human coronavirus; hMPV, human metapneumovirus; HR, hazard ratio; HRV, human rhinovirus; IRR, incidence rate ratio; LRTI, lower respiratory tract infection; NR, not reported; OR, odds ratio; PCV, pneumococcal conjugate vaccine; PIV, parainfluenza virus; PV, picornavirus; RR, risk ratio; RSV, respiratory syncytial virus; RTI, respiratory tract infection; SARI, severe acute respiratory infection; SARS-CoV-2, severe acute respiratory syndrome coronavirus 2; US, United States.

^a^The study measures vaccine efficacy.

^b^Different outcomes measured in the same RCT population.

^c^PCV7 + influenza vaccine versus influenza vaccine alone.

^d^The study measures vaccine effectiveness (See Methods section).

Eleven of 16 manuscripts were graded as having a risk of bias, mostly due to a lack of information about the sampling method, randomization, allocation, the ascertainment of vaccination status, the adjustment for confounding factors, or the microbiology method used for diagnosing the viral RTI (Supplementary Table 6).

### PCV Effects Against Viral RTI in Children

**Figure 2.**
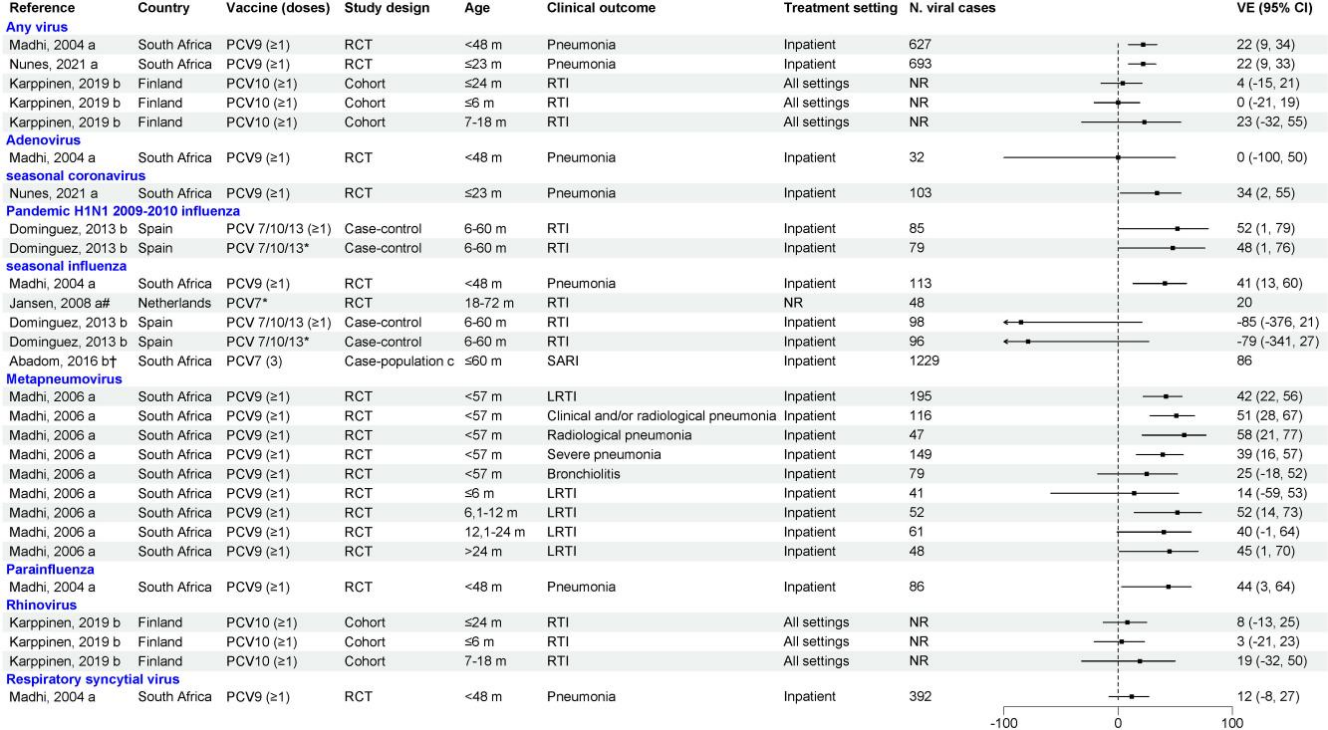
Efficacy/effectiveness of pneumococcal conjugate vaccines (PCVs) against viral respiratory tract infection in children. “a” indicates efficacy; “b” indicates effectiveness; "c" similar to screening design. *Received all recommended doses for age. †Crude vaccine effectiveness calculated as 1 – case-population ratio × 100% given by the article. #PCV7 + influenza vaccine compared to influenza vaccine + placebo in participants who received all recommended doses for age. Abbreviations: CI, confidence interval; LRTI, lower respiratory tract infection; NR, not reported; PCV, pneumococcal conjugate vaccine; RCT, randomized controlled trial; RTI, respiratory tract infection; SARI, severe acute respiratory infection; VE, vaccine effectiveness.

PCV direct effects against viral RTI in children have been evaluated in 2 RCTs in South Africa (PCV9) and the Netherlands (PCV7) and 3 observational studies in Finland (PCV10), Spain (PCV7/10/13), and South Africa (PCV7) (Figure 2). The PCV9 RCT showed efficacy ranging from 22% to 51% against hospitalized pneumonia due to any virus, influenza, parainfluenza virus, human seasonal coronaviruses, and hMPV. No protection was observed against adenovirus or RSV. An RCT in the Netherlands showed that PCV7 was effective against seasonal influenza RTI (VE, 20%) [18]. A PCV10 cohort study in Finland did not find any effectiveness against RTI due to any virus or rhinovirus [20].

A case-control study conducted in Spain showed that receipt of any PCV (PCV7, PCV10, or PCV13) was effective against hospitalized influenza caused by H1N1 pandemic influenza but not for seasonal influenza during 2010–2011 [29]. Finally, a case-population study conducted in South Africa showed that receipt of PCV7 reduced the risk of influenza-associated severe acute respiratory illness [19].

A subset of studies also evaluated the direct effects of PCV against viral RTI in children by risk group (Supplementary Table 7). In the PCV9 RCT, when analysis was stratified by human immunodeficiency virus (HIV) status, VE was generally only observed in HIV-uninfected children, with the exception of hMPV [11, 17, 21]. In the Spanish case-control study that evaluated PCV VE against influenza hospitalizations, no protection was observed in children with underlying diseases such as chronic heart or lung disease [29].

The overall effect of PCV against viral RTI among children <5 years old was described in 6 manuscripts, all of which compared incidence rates before and after PCV introduction. Results varied by virus, design, age, and population, and most 95% CIs were wide (Supplementary Table 8) [22–24, 26, 27]. A study conducted in Western Australia found declines of pneumonia hospitalizations associated with influenza A, parainfluenza virus, and RSV (IRR <1) for some age groups, primarily in non-Aboriginal children [24]. In contrast, there was no clear evidence for declines in influenza-associated hospitalizations following PCV introduction in Iceland and Mongolia as CIs were wide and included 1 [26, 27]. For RSV, in addition to the Western Australia study, 2 large studies in the United States (US) reported declines in RSV-coded hospitalizations following PCV7 introduction, but no reduction in RSV-associated hospitalization was seen in Mongolia and Australia's Northern Territory following PCV introduction [22–25, 27].

### PCV Effect Against Viral LRTI in Adults

**Figure 3.**
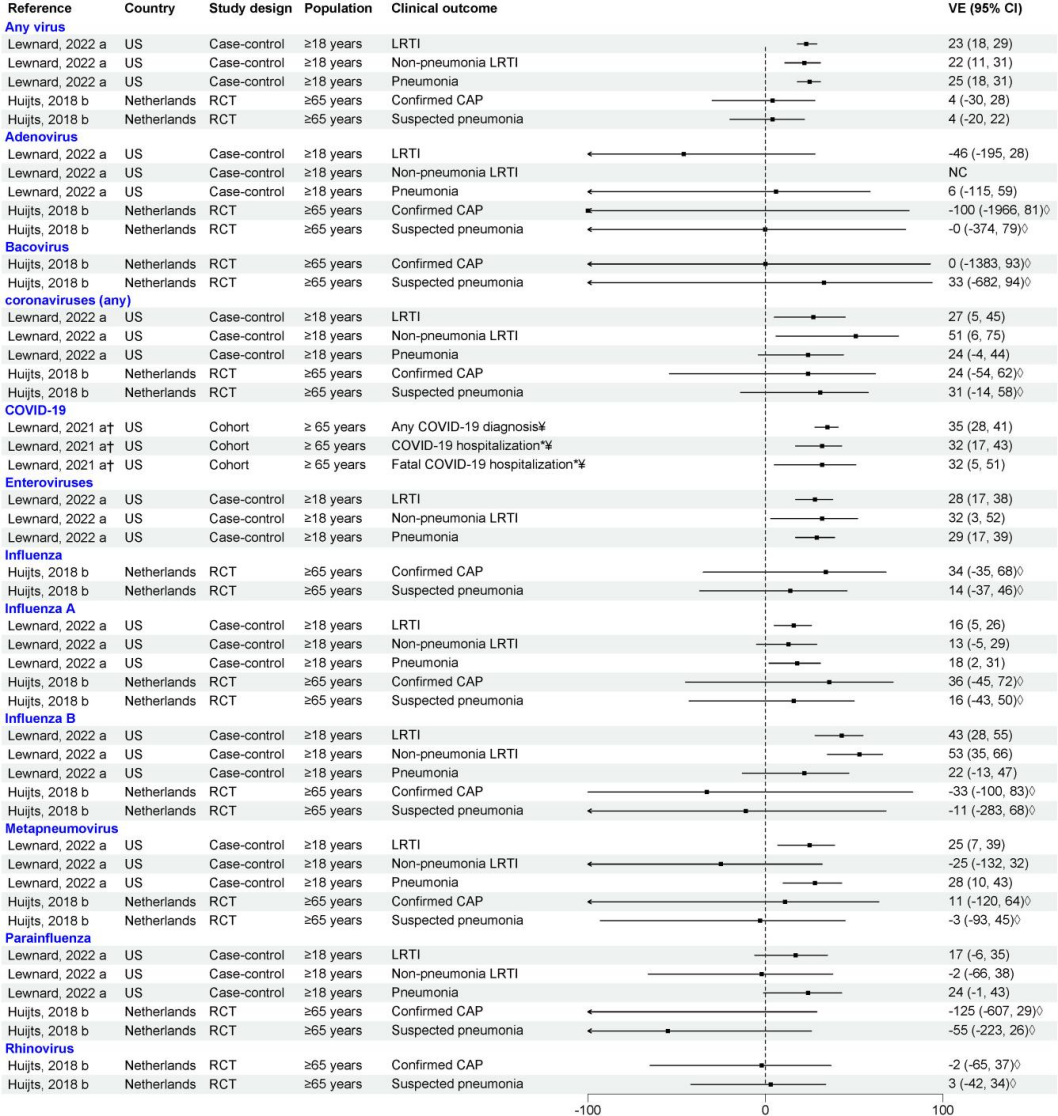
Efficacy/effectiveness of 13-valent pneumococcal conjugate vaccine against viral lower respiratory tract infection in adults (outpatient and inpatient). “a” indicates effectiveness; “b” indicates efficacy. †Crude vaccine effectiveness calculated from data provided by the article. *Only inpatient setting. ◊99.3% confidence interval. ¥COVID-19 cases were diagnosed either clinically or with a stand-alone severe acute respiratory syndrome coronavirus 2 test. Abbreviations: CAP, community-acquired pneumonia; CI, confidence interval; COVID-19, coronavirus disease 2019; LRTI, lower respiratory tract infection; NC, noncomputable; RCT, randomized controlled trial; US, United States; VE, vaccine effectiveness.

Three studies examined the direct effects of PCV13 against viral LRTI in adults: one RCT conducted in the Netherlands and 2 observational studies in the US [14, 28, 30] (Figure 3). In the Netherlands RCT, PCV13 did not display efficacy against confirmed CAP due to any viruses (VE, 3.6% [99.3% CI, −29.6% to 28.3%]). Point estimates for human coronaviruses and influenza A were consistent with protection, although conservative 99.3% CIs were wide and spanned zero. In a case-control study in the US, Lewnard and colleagues evaluated VE of PCV13 against different LRTI outcomes (LRTI, pneumonia, and nonpneumonia LRTI) due to different respiratory viruses. PCV13 effectiveness ranged between 22% and 25% for all 3 LRTI outcomes for any virus. Consistent PCV13 protection was observed against different LRTI outcomes due to seasonal coronavirus (VE 24%–51%), enteroviruses (VE 29%–32%), and influenza A and B (VE 13%–53%), with some CIs spanning zero [14]. Results varied by outcome for hMPV and parainfluenza, and no protection was found for adenovirus or RSV. A cohort study conducted in US adults prior to coronavirus disease 2019 (COVID-19) vaccine availability reported PCV13 effectiveness of 35% against COVID-19 diagnosis, 32% against COVID-19 hospitalization, and 32% against fatal COVID-19 hospitalization in adults aged ≥65 years [30].

## DISCUSSION

Results from this systematic review are generally consistent with PCVs conferring protection beyond etiologically confirmed vaccine-type pneumococcal disease and more specifically, protection against RTIs ascribed to respiratory viruses. Our findings are complemented by a growing body of evidence [31], demonstrating that PCVs afford protection against all-cause pneumonia and LRTI beyond that estimated from vaccine serotype pneumococcal CAP incidence and VE against this outcome.

Substantial evidence exists for interactions between respiratory viruses and bacteria in LRTI. This review summarizes the evidence that PCVs confer protection in children and adults against LRTIs involving viral pathogens. The strongest evidence stems from studies investigating PCV VE against viral LRTIs in children, and for influenza in particular [32, 33]. For seasonal coronaviruses, results from studies in adults and children were generally supportive of a protective effect of PCV, with potential mechanisms discussed in a recent minireview [32]. Evidence was suggestive but not as robust for protection among adults, and for RTI due to any viruses, parainfluenza virus, and hMPV. Results for RSV were mixed. Where evaluated, there was consistent evidence that PCVs do not provide any protection against rhinovirus or adenoviruses.

Differences between studies in terms of vaccine (type, schedule, and coverage), study design, RTI outcome, sample size, and populations likely explain some heterogeneity in the results in children. For instance, a small prospective cohort study in Finland found no PCV10 effectiveness against viral RTI in inpatient and outpatient settings [20], as opposed to 22% efficacy of PCV9 against hospitalized viral LRTI measured in a large RCT in South Africa [11, 21]. However, the Finnish study also reported an effectiveness of 12% against all RTI episodes [20]. Half of the observational studies did not provide vaccine coverage rates. When reported, coverage rates in the included studies were generally high, ranging from 71% to 97%, but with substantial year-to-year variation. This makes interpretation of the influence of vaccine coverage on protection against viral RTI difficult. Likewise, most of the studies assessed the vaccine effectiveness of ≥1 dose without stratification per number of doses received, which prevented us to assess the influence of different schedules on the VE estimates.

Although evidence in adults was more limited, directionally results were consistent with data from children, with protection reported for LRTI associated with influenza and coronaviruses, including COVID-19. Additionally, the lack of effect against adenoviruses was consistent between adults and children. Reductions in viral-associated LRTI might help explain observed reductions in all-cause pneumonia in adults. In 4 observational studies and one RCT, PCV13 VE against all-cause pneumonia ranged between 6% and 12% in older adults [12, 13, 34–36]. This reduction is greater than what would be expected solely due to reductions in etiologically confirmed, PCV13-type CAP (usually identified by serotype-specific urinary antigen detection [UAD] assays), estimated by multiplying PCV13 VE against vaccine-type pneumococcal CAP by the percentage of CAP due to PCV13 serotypes. As one example, a large multicenter US study reported that 4.2% of any pneumonia was due to PCV13 serotypes [37]. That proportion multiplied by a VE against vaccine-type CAP of 45.6% [38] would predict a reduction of 1.9% in all-cause pneumonia. By contrast, 3 observational studies in the US reported PCV13 VE against all-cause CAP of 7%–10% [13, 35]. Our results raise the possibility that the additional reduction in burden may occur in part because of reductions in virus-associated LRTI.

Several possible explanations exist for the reported effects of PCVs on viral LRTIs. The simplest explanation is that PCVs are preventing pneumococcal–viral coinfections; however, due to differential sensitivities of etiologic tests (eg, culture, UADs, and polymerase chain reaction) for bacteria and viruses [39, 40], only the viral component of the coinfection is detected. This effect may be enhanced by antibiotic treatment prior to specimen collection, which will reduce the ability to culture *Streptococcus pneumoniae* but will not affect virus detection rates [41]. Other potential explanations are related to *S pneumoniae*–virus interactions. For example, PCVs, by modulating the serotype distribution in carriage or influencing the upper airway microbiome [42, 43], may modify host susceptibility to viral LRTIs. Some support exists for this hypothesis. Pneumococcal carriage at the time of viral infection has been associated with an impairment of virus-specific immune responses to influenza and severe acute respiratory syndrome coronavirus 2 (SARS-CoV-2) [9, 44]. Pneumococcal carriage and density have been associated with increased odds of SARS-CoV-2 infection in adults [33]. Viral infection itself may increase pneumococcal colonization density, which may increase the risk for subsequent RTIs in children [10, 45]. In adults, PCVs not only prevent carriage acquisition but also help to control carriage density [46]. PCVs could thus reduce the risk of viral LRTIs if pneumococcus is a component of a viral RTI causal chain. If these latter hypotheses are confirmed, our data suggest that this interaction varies by virus and—since carriage prevalence generally returns to baseline post-PCV introduction—by pneumococcal serotype, the latter supported by a study of pneumococcal and RSV interaction from Israel [47]. The various hypotheses are not mutually exclusive, and each could contribute to the reduction of viral LRTI following PCV immunization. Similarly, discrepancies in etiologic fractions of pneumonia caused by *S pneumoniae* as determined by vaccine probe studies compared to diagnostic studies may be caused by incomplete testing or limited test sensitivity, but may also suggest alternative disease prevention pathways.

To our knowledge, no other systematic review has explored the effect of PCVs on viral LRTI. The strengths of this review are the inclusion of 2 global databases and grey literature, and a broad period of included studies (2000–2022), from the introduction of PCV7 onward. However, the interpretation of our findings warrants caution due to the limited number of studies identified, particularly for adults and for risk groups. Few included studies were assigned a low risk of bias (n = 4 [22%]), and most data from clinical trials originated from post hoc analysis. The limited number of studies per virus (except for influenza) precludes us from drawing robust conclusions. The heterogeneity in outcomes, population, vaccine schedules, study designs, and settings and limited sample size limit the generalizability of our findings.

Our findings enabled us to identify future research needs, such as larger studies of direct effects of PCV against RTI due to specific viruses. Only one study evaluated the direct effect of PCV on adenovirus [11], a common viral infection in children [48]. For RSV, 4 of 5 pediatric studies demonstrated population-level impact of PCV7 and PCV13 on RSV-related hospitalizations [22–24, 27, 31], but the South African PCV9 RCT found no efficacy [11]. However, substantial year-to-year variations in RSV circulation make interpretation difficult [22–25, 27]. Given the importance of RSV as a major cause of LRTI, particularly in early infancy, and the development of new vaccines and therapeutics, further research into the interplay between pneumococcus and RSV and the role of vaccination is warranted [49]. The most supportive evidence in adults was seen for coronaviruses; however, these studies were conducted prior to the availability of COVID-19 vaccines, so it would be interesting to explore whether PCV extends the protection provided by COVID-19 vaccination. Additional clinical and translational research is needed to understand the mechanisms underpinning PCV protection against viral LRTIs. Future studies should explore the interactions between viruses, pneumococci, the upper respiratory microbiome, and mucosal immunity. Finally, future studies will have the advantage of evaluating the effect of newer, higher-valency PCVs such as PCV15 and PCV20 on viral respiratory outcomes.

The importance of these cumulative observations and their potential impact on public health warrant additional well-controlled studies with prespecified endpoints. Given the availability of several safe and effective PCVs, RCTs are not ethical or feasible to conduct in most settings. However, real-world evidence is increasingly being used to document additional clinical benefits of vaccination that were not estimated during clinical development programs [50]. Moving forward, evidence generated from well-controlled, real-world studies should be considered by regulatory agencies and vaccine technical committees when assessing both the clinical benefits and broader public health impact of vaccines at a population level. Additional future research could also evaluate the impact of viral vaccines on confirmed bacterial pneumonias.

## CONCLUSIONS

Available studies suggest that PCVs confer protection against viral RTI, with the most robust data from children. While data from adults were more limited, they were consistent with results in children for the most part, adding confidence that reported findings represent a true biological effect. If results from studies conducted to date are corroborated, PCV protection against RTIs beyond those due to pneumococci might better capture the full spectrum of public health benefits of PCVs, thereby informing vaccine policymaking and economic evaluations.

## Supplementary Data

Supplementary materials are available at *The Journal of Infectious Diseases* online (http://jid.oxfordjournals.org/). Supplementary materials consist of data provided by the author that are published to benefit the reader. The posted materials are not copyedited. The contents of all supplementary data are the sole responsibility of the authors. Questions or messages regarding errors should be addressed to the author.

## Supplementary Material

jiae125_Supplementary_Data
