# Supplementary material for: Effect of Pneumococcal Conjugate Vaccines on Viral Respiratory Infections: A Systematic Literature Review
Source: J Infect Dis. 2024 Mar 11;230(3):e657–67. doi: 10.1093/infdis/jiae125 (PMC11420806; doi:10.1093/infdis/jiae125)
Supplement: jiae125_Supplementary_Data [file jiae125_supplementary_data.docx]

Supplemental files to:

Effect of pneumococcal conjugate vaccines on viral respiratory infections: a systematic literature review

Table of Contents

[**Supplementary Table 1.** Serotypes included in pneumococcal conjugate vaccines used in studies included in this systematic literature review. 2](#_Toc153789566)

[**Supplementary Table 2.** PICOTS inclusion and exclusion criteria 3](#_Toc153789567)

[**Supplementary Table 3.** Search strategy 4](#_Toc153789568)

[**Supplementary Table 4.** Excluded studies after full-text review, with the reason for exclusion. 5](#_Toc153789569)

[**Supplementary Table 5.** Clinical outcome definitions for included studies. 6](#_Toc153789570)

[**Supplementary Table 6.** Summary of risk of bias assessment. 8](#_Toc153789571)

[**Supplementary Table 7.** Efficacy/Effectiveness of PCV13 vaccination against viral respiratory tract infection in inpatient children by presence of risk factors. 13](#_Toc153789572)

[**Supplementary Table 8.** Overall effect of PCVs on viral RTI in children (inpatient setting). 15](#_Toc153789573)

## **Supplementary Table 1. Serotypes included in pneumococcal conjugate vaccines used in studies included in this systematic literature review.**

| **PCV** | **Serotypes included** |
| --- | --- |
| **PCV7** | **4, 6B, 9V, 14, 18C, 19F, and 23F** |
| **PCV9^a^** | **PCV7 + serotypes 1, and 5** |
| **PCV10** | **PCV9 + serotype 7F** |
| **PCV13** | **PCV10 + serotypes 3, 6A, and 19A.** |

**PCV: Pneumococcal conjugated vaccine**

**^a^ unlicensed vaccine used in clinical trials**

## **Supplementary Table 2. PICOTS inclusion and exclusion criteria**

| Parameter | Criteria |
| --- | --- |
| Population | **Included:**  **Children (<18 year) and adults (≥18 years), of any gender.** |
| Intervention | **Included: Vaccination with PCV7, PCV9, PCV10 or PCV13, as measured by** PCV (PCV7, PCV9, PCV10, PCV13)-specific efficacy/effectiveness against, or impact on, virus-related URTIs in children; and PCV-specific efficacy/effectiveness against, or impact on, virus-related LRTIs in children and adults.  **Excluded:**  Studies with patients receiving influenza vaccine in addition to PCV only in the interventional/exposed group. (Not possible to distinguish the effect of each vaccine). |
| Comparison | Depending on study design, the comparator may include non-PCV-vaccinated arms in clinical trials, or non-PCV-vaccinated population in observational studies. |
| Time | Studies published from 2000 to June 2022 |
| Study design | **Included:**  **-** Randomized controlled trials and observational studies.  - Online reports/white papers (if available and relevant)  **Excluded:**   - Publications of the following type will be excluded:   - case reports   - editorials   - commentaries   - narrative reviews - Non-human data (e.g., in-vitro, in-silico, animal models) will be excluded. |
| Outcome | - Respiratory tract infections (RTIs) of viral etiology (single or co-infection), including LRTIs (bronchitis, bronchiolitis, pneumonia) and URTIs (otitis media, acute otitis media, sinusitis, others) in children |
| Country | Studies from all countries in the world are eligible |
| Language | Included: English, French, Spanish, Portuguese. |

PCV: pneumococcal conjugate vaccine; LRTI: lower respiratory tract infection; RTI: respiratory tract infection; URTI: upper respiratory tract infection.

## **Supplementary Table 3. Search strategy**

| **Search string** | **No. of hits (duplicates excluded)** |
| --- | --- |
| ((((acute lower respiratory tract infection) OR ("CAP") OR (community acquired pneumonia) OR ("LRTI") OR ("LRTIs") OR ("URTIs") OR ("URTI") OR (upper respiratory tract infection) OR (bronchitis) OR (bronchiolitis) OR (pneumonia) OR (pleural effusion) OR (acute respiratory infection) OR (COVID-19) OR ("coronavirus disease 2019") OR ("respiratory infections") OR (respiratory tract infections) OR ("RTI") OR ("RTIs") OR (otitis media) OR (rhinitis) OR (tonsillitis) OR (pharyngitis) OR (laryngitis) OR (sinusitis) OR ("AOM") OR ("OM"))  AND ((pneumococcal conjugate vaccination ) OR (pneumococcal conjugate immunization*) OR (pneumococcal conjugate immunisation*) OR (pneumococcal conjugate vaccine) OR (pneumococcal conjugate vaccines) OR (PCV7) OR (PCV) OR (PCV9) OR (nine-valent PCV) OR (13-valent PCV) OR (PCV13) OR (13-valent conjugate vaccine) OR (13-valent pneumococcal conjugate vaccine) OR (9-valent pneumococcal conjugate vaccine) OR (9-valent PCV) OR (seven-valent pneumococcal conjugate vaccine) OR (PCV10) OR (PHiD-CV10) OR (PCV-7) OR (PCV-13) OR (PCV-10) OR (PHiD-CV) OR ("pneumococcal non-typeable Haemophilus influenzae protein D-conjugate vaccine") OR (11-valent PCV) OR (PCV11) OR (11-valent conjugate vaccine) OR (eleven-valent pneumococcal conjugate vaccine)))  AND ((Influenza) OR (virus) OR (viral) OR (severe acute respiratory illness) OR ("SARI") OR ("influenza-like illness") OR ("ILI") OR ("viral pneumonia") OR ("viral pathogens") OR (Respiratory syncytial virus) OR ("RSV") OR (Influenza virus) OR (Influenza virus A) OR (Influenza virus B) OR (adenovirus) OR (adenoviruses) OR (Influenza viruses) OR (parainfluenza virus) OR (parainfluenza viruses) OR ("PIV (1-4)") OR ("PIV (1-3)") OR (metapneumovirus) OR (metapneumoviruses) OR ("hMPV") OR ("HMPV") OR (picornavirus) OR (picornaviruses) OR ("viral associated") OR ("Influenza-associated") OR ("virus associated") OR (Parainfluenza) OR (bocavirus) OR (bocaviruses) OR (coronavirus) OR (coronaviruses) OR (SARS-CoV-2) OR (rhinovirus) OR ("HCoV") OR ("HCOV") OR ("CoV-299E") OR ("HCoV-HKU1") OR ("HCoV-NL63") OR ("HCoV-OC43") OR ("SARS-CoV") OR ("MERS-CoV") OR (enterovirus) OR (enteroviruses) OR ("respiratory viruses") OR ("respiratory virus") OR ("hMPV-associated") OR ("coronavirus-associated") OR ("CoV") OR ("CoVs")))  AND (("2000/01/01"[Date - Publication]: "3000"[Date - Publication])) | **1,665** |

## **Supplementary Table 4. Excluded studies after full-text review, with the reason for exclusion.**

| **Reference** | **Reason for exclusion** |
| --- | --- |
| Abdulah, 2021 | Poor quality of the study, compared countries with PCV universal coverage and countries without universal coverage by number of vaccine doses administered; did not fit completely with the selection criteria. |
| Chang, 2016 | The measure reported was change in proportions |
| Elemraid, 2013 | Only included proportions; measure of non-interest for the review |
| Gentile, 2015 | Only included proportions; measure of non-interest for the review |
| Hanafy, 2022 | Patients received influenza vaccine in addition to PCV in the exposed group. Not possible to examine the effect of PCV alone. |
| Haro, 2017 | Measure reported was change in proportions |
| Jimenez, 2017 | Only included proportions; measure of non-interest for the review |
| Kubale, 2021 | They did not compare the incidence of influenza-related pneumonia across cohorts, but the odds of pneumonia following influenza not the viral pneumonia itself |
| Marom, 2017 | Only included proportions; measure of non-interest for the review |
| Muhandule, 2020 | Reported on carriage (in healthy children) as clinical outcome, out of scope. |
| Shin, 2018 | Only included proportions; measure of non-interest for the review |
| Simonsen, 2011 | Only included changes in modeled viral pneumonia rate per change in PCV7 uptake. No VE nor impact. |
| Vestjens, 2017 | Only included proportions; measure of non-interest for the review |
| Weinberger, 2013 | Wrong outcome (radiologically confirmed alveolar pneumonia not specifically due to RSV). RSV data not compared between pre- and post-vaccine periods. |

## **Supplementary Table 5. Clinical outcome definitions for included studies.**

| **Study** | **Clinical outcome** | **Definition** |
| --- | --- | --- |
| Madhi, 2004 | Pneumonia | Children were diagnosed as having “pneumonia” in the presence of WHO-AC, or if the child fulfilled the clinical diagnosis of LRTI without wheezing on chest auscultation, but with rales and/or bronchial breathing. WHO-AC: The presence of a dense opacity that may be a fluffy consolidation of a portion or whole of a lobe or of the entire lung, often containing air bronchograms and sometimes associated with pleural effusion or a pleural effusion in the lateral pleural space associated with a pulmonary infiltrate / or an effusion large enough to obscure such an opacity. |
| Madhi, 2006 | LRTI | All children hospitalized with a study physician diagnosis of pneumonia or bronchiolitis, irrespective of symptoms found by clinical examination or chest radiography (CXR) |
|  | Clinical pneumonia | Children that had evidence of alveolar consolidation on CXR (CXR-AC) or if they fulfilled the clinical diagnosis of LRTI without wheezing on chest auscultation but had rales and/or bronchial breathing. |
|  | WHO-confirmed radiological pneumonia | The presence of a dense opacity that may be a fluffy consolidation of a portion or whole of a lobe or of the entire lung, often containing air bronchograms and sometimes associated with pleural effusion or a pleural effusion in the lateral pleural space associated with a pulmonary infiltrate / or an effusion large enough to obscure such an opacity. |
|  | Bronchiolitis | Children with the presence of wheezing on chest auscultation performed by one of the study doctors and in the absence of documented alveolar consolidation on chest radiograph or bronchial breathing on chest wall auscultation. |
|  | WHO-Severe pneumonia | The children had a cough <14 days in duration and lower chest wall in-drawing and/or any of the following signs and symptoms of severe pneumonia: feeding difficulties, convulsions, central cyanosis, or encephalopathy |
| Jansen, 2008 | RTI | Diagnosis registered according to the International Classification of Primary Care: acute otitis media (AOM) (H71); cough (with fever) (R05); acute upper RTI (R74), including common cold and pharyngitis; sinusitis (R75); acute tonsillitis (R76); acute laryngitis/tracheitis (R77); acute bronchitis/bronchiolitis (R78); influenza (R80); pneumonia (R81); pleurisy/pleural effusion (R82); and other respiratory infection (R83) |
| Dominguez, 2013 | RTI | Patient between 6 months and 5 years of age admitted to hospital for >24 hours with influenza virus infection confirmed by real-time reverse-transcription polymerase chain reaction. |
| Foote, 2015 | LRTI | All hospital discharge records with an LRTI listed as any one of up to 15 diagnoses. The ICD-9 codes considered were: pulmonary tuberculosis (011), pulmonary anthrax (022.1), pulmonary diseases caused by Mycobacterium (031.0), whooping cough (033), respiratory syncytial virus (RSV) (079.6), syphilis of lung (095.1), acute bronchitis (466.0), acute bronchiolitis (466.1), pneumonia (480-486), influenza (487), influenza due to identified 2009 H1N1 influenza virus (488.1), empyema (510), pleurisy with effusion (511.1), abscess of lung and mediastinum (513), rheumatic pneumonia (517.1) and congenital pneumonia (770.0) (2,3). Acute bronchiolitis, pneumonia, RSV (466.11, 480.1 and 079.6) and influenza (487 and 488.1) |
| Weinberger, 2015 | LRTI | Diagnostic discharge codes listed anywhere in the patient’s discharge record, including pneumococcal pneumonia/lobar pneumonia (International Classification of Diseases, Ninth Revision [ICD-9], code 481), pneumococcal septicemia (038.2), respiratory syncytial virus (079.6, 466.11, 480.1), influenza (487.0–487.9), and bronchiolitis (466). |
| Abadom, 2016 | Severe acute respiratory infection | WHO case definition for severe acute respiratory infection: an acute respiratory illness with a history of fever or measured fever of ≥38 °C and cough, with onset within the past 10 days, requiring hospitalization. |
| Fathima, 2018 | Pneumonia | ICD-9-CM codes (003.22, 031.0, 052.1, 055.1, 112.4, 115.05, 115.15, 115.25, 136.3, 480-486), ICD-10-AM codes (J10- J11, J12-J18, B01.2, B05.2, B37.1, B59) |
| Karppinen, 2019 | RTI | An episode of acute RTI was defined as rhinitis, cough, or wheezing, with or without fever, documented in the diary by the parents, or by a physician as a diagnosis of an acute RTI. |
| Binks, 2020 | Pneumonia | ICD-10 code J09-J18·9. |
| Sigurdsson, 2020 | Pneumonia | ICD-10 discharge diagnosis of J09-J18. |
| Nunes, 2021 | Pneumonia | Defined as presence of alveolar consolidation on chest X-ray, or clinical diagnosis of LRTI without wheeze on chest auscultation, but with rales and/or bronchial breathing. |
| Do, 2022 | Pneumonia | An adapted WHO pneumonia case definition (Not specified). Also  with an arterial O2 saturation<93%, and radiological confirmed pneumonia. |
| Huijts, 2018 | CAP | ‘Confirmed CAP’ was defined as an episode with a chest X-ray consistent with pneumonia together with the presence of two or more of the following clinical criteria: cough, purulent sputum, temperature >38.0°C or 10 109 white blood cells/litre or >15% bands), C-reactive protein more than three times the upper limit of normal or hypoxaemia (oxygen pressure <60 mmHg while the patient was breathing room air). Patients with ‘suspected pneumonia’ were all individuals presenting with a clinical suspicion of pneumonia at a participating sentinel centre, which also included those with ‘confirmed CAP’ |
| Lewnard, 2021 | Covid-19 diagnosis | COVID-19 diagnosis, defined as a positive result of a molecular test for SARS-CoV-2 infection or a clinically confirmed COVID-19 diagnosis |
|  | COVID-19 hospitalization | COVID-19 hospitalization, defined as a new inpatient admission between 7 days before and 28 days after a COVID-19 diagnosis |
| Lewnard, 2022 | LRTI | Cases were individuals receiving any LRTI diagnosis according to International Classification of Diseases, 10th revision, clinical modification (ICD-10-CM) codes during the study period who tested positive for any of the study endpoint viral infections: influenza A and B 61 viruses, RSV, HCoVs (including the 229E, HKU1, NL63, and OC43 subtypes), parainfluenza viruses 62 (types 1-4), adenoviruses, human metapneumovirus (HMPV), and enteroviruses (including rhinoviruses) |
|  | Non-pneumonia LRTI | Cases were individuals receiving LRTI cases without pneumonia diagnoses according to International Classification of Diseases, 10th within 30 days following an initial no pneumonia LRTI diagnosis |
|  | Pneumonia | Cases were individuals receiving the diagnosis according to International Classification of Diseases, 10th revision, clinical modification (ICD-10-CM) codes. |

CXR: chest radiography; AC: alveolar consolidation; ICD-9: international Classification of Diseases 9; RTI: respiratory tract infection; LRTI:lower respiratory tract infection, WHO: World Health Organization.

## **Supplementary Table 6. Summary of risk of bias assessment.**

**Please refer to supplementary tables 6a, 6b, 6c, and 6d for risk of bias assessment for individual studies.**

|  | | |
| --- | --- | --- |
| **Study design** | **Studies at risk of bias** | **Total** |
| Prospective observational cohort studies | 0 (0%) | 2 |
| Case-control studies | 1 (33%) | 3 |
| Pre- and post-studies | 5 (83%) | 6 |
| Randomized controlled studies | 5 (100%) | 5 |
| Total | 11 (69%) | 16 |

Supplementary table 6a. Risk of bias assessment of cohort studies included.

| Author, journal | Reference in manuscript | Selection | | | Comparability | Outcome | |  |
| --- | --- | --- | --- | --- | --- | --- | --- | --- |
|  |  | Representativeness of the PCV vaccinated cohort | Selection of the non-vaccinated cohort | Ascertainment of PCV vaccination | Comparability of cohorts on the basis of the design or analysis | Assessment of viral respiratory disease | Adequacy of follow up of cohorts | Did the measures provided have 95%IC for the pre-to-post changes? |
| Karppinen, Vaccine 2019;37:2935-41. | 18 | 1 | 1 | 1 | 1 | 1 | 1 | 1 |
| Lewnard, J Infect Dis 2022;225:1710-20. | 30 | 1 | 1 | 0 | 2 | 1 | 1 | 1 |

95%IC – 95% confidence interval

Supplementary table 6b. Risk of bias assessment of case-control studies included.

| Author, journal | Reference in manuscript | Selection | | | | Comparability | Exposure | |  |
| --- | --- | --- | --- | --- | --- | --- | --- | --- | --- |
|  |  | Is the case (viral respiratory disease) definition adequate? | Representativeness of the cases | Selection of Controls | Definition of Controls | Comparability of cases and controls on the basis of the design or analysis | Ascertainment of PCV vaccination | Same method for ascertainment PCV vaccination status for cases and controls. | Did the measures provided have p values or 95%IC for the pre-to-post changes? |
| Abadom, Vaccine 2016;34:5649-55. | 21 | 1 | 1 | 1 | 1 | 1 | 0 | 0 | 1 |
| Dominguez, Pediatr Infect Dis J 2013;32:330-4. | 19 | 1 | NR | 0 | 1 | 2 | 1 | 1 | 1 |
| Lewnard, J Infect Dis 2022;227:498-511. | 14 | 1 | 1 | 1 | 1 | 2 | 1 | 1 | 1 |

NR – not reported, 95%IC – 95% confidence interval

Supplementary table 6c. Risk of bias assessment of before and after studies included.

| Author, journal | Reference in manuscript | Were eligibility/selection criteria for the study population prespecified and clearly described? | Were study participants representative of those eligible for PCV? | Was the PCV uptake clearly described in the study population? | Was viral respiratory infection disease defined and assessed? | Did the measures provided have 95%IC for the pre-to-post changes? |
| --- | --- | --- | --- | --- | --- | --- |
| Binks, Lancet Child Adolesc Health 2020;4:425-34. | 23 | Yes | Yes | Yes | NR | Yes |
| Do, 12th ISPPD, 2022. | 28 | Yes | Yes | NI | Yes | Yes |
| Fathima, Clin Infect Dis 2018;66:1075-85. | 25 | Yes | No | No | Yes | No |
| Foote EM, Int J Circumpolar Health 2015;74:29256. | 27 | Yes | Yes | Yes | NR | Yes |
| Sigurdsson, Vaccine 2020;38:2707-14. | 24 | Yes | Yes | Yes | Yes | Yes |
| Weinberger, PLoS medicine 2015;12:e1001776. | 26 | No | Yes | No | NR | Yes |

NI – not interpretable; NR – not reported, 95%IC – 95% confidence interval

Supplementary Table 6d. Risk of bias assessment of control trials studies included.

| Author, journal | Reference in manuscript | Domain 1 | | | | Domain 2 | | | | | Domain 3 | | | Domain 4 | | | Overall RoB | |
| --- | --- | --- | --- | --- | --- | --- | --- | --- | --- | --- | --- | --- | --- | --- | --- | --- | --- | --- |
|  |  | Randomization process | | | Final | Deviations from intended interventions | | | | Final | Missing outcome data | | Final | Outcome measurement | | Final | |  |
|  |  | 1.1 | 1.2 | 1.3 |  | 2.1 | 2.2 | 2.3 | 2.4 |  | 3.1 | 3.2 |  | 4.1 | 4.2 |  | |  |
| Huijts, Clin Microbiol Infect 2018;24:764-70. | 29 | NI | NI | NI | Some concerns | No | NI | Yes | NA | Some concerns | Yes | Yes | Low risk | No | No | Low risk | | Some concerns |
| Jansen, J Pediatr 2008;153:764-70. | 20 | NI | NI | No | Some concerns | No | No | Yes | NA | Low risk | Yes | NA | Low risk | No | No | Low risk | | Some concerns |
| Madhi, J Infect Dis 2006;193:1236-43. | 22 | NI | NI | Yes | Some concerns | No | NI | Yes | NA | Some concerns | Yes | NI | Some concerns | No | No | Low risk | | High risk |
| Madhi, Nat Med 2004;10:811-3. | 11 | NI | NI | N | Some concerns | No | NI | Yes | NA | Some concerns | NI | NA | high risk | No | No | Low risk | | High risk |
| Nunes, mBio 2021;12. | 17 | NI | NI | No | Some concerns | N | NI | Yes | NA | Some concerns | NI | NI | high risk | No | No | Low risk | | High risk |

1.1 Was the allocation sequence random? 1.2 Was the allocation sequence concealed until participants were enrolled and assigned to interventions? 1.3 Did baseline differences between intervention groups suggest a problem with the randomization process? 2.1 Were participants aware of their assigned intervention during the trial? 2.2 Were carers and people delivering the interventions aware of participants' assigned intervention during the trial? 2.3 Was an appropriate analysis used to estimate the effect of assignment to intervention? 2.4 If N/PN/NI to last question: Was there potential for a substantial impact (on the result) of the failure to analyze participants in the group to which they were randomized? 3.1 Were data for this outcome available for all, or nearly all, participants randomized? 3.2 If N/PN/NI to last question: Is there evidence that the result was not biased by missing outcome data? 4.1. Was the method of measuring the viral respiratory disease inappropriate? 4.2. Could measurement or ascertainment of the viral respiratory disease have differed between intervention groups?

RoB – Risk of Bias; PY – probably yes; PN – probably no; NI – not interpretable; NA – not assessed

## **Supplementary Table 7.** Efficacy/Effectiveness of PCV13 vaccination against viral respiratory tract infection in inpatient children by presence of risk factors.

| **Reference** | **Vaccine exposure (n. doses)** | **Population◦** | **Clinical outcome** | **VE value** |  |
| --- | --- | --- | --- | --- | --- |
|  |  |  |  |  |  |
| **Any virus** | | | | |  |
| Madhi, 2004 a | PCV9 (≥1) | <48 m, HIV infected | Pneumonia | 23 (-4 to 43) |  |
| Madhi, 2004 a | PCV9 (≥1) | <48 m, HIV uninfected | Pneumonia | 22 (6 to 35) |  |
| Nunes, 2021 a | PCV9 (≥1) | ≤23 m, HIV infected | Pneumonia | 17 (-5 to 34) |  |
| Nunes, 2021 a | PCV9 (≥1) | ≤ 23 m, HIV uninfected | Pneumonia | 25 (9 to 38) |  |
| **Adenovirus** | | | | |  |
| Madhi, 2004 a | PCV9 (≥1) | <48 m, HIV infected | Pneumonia | -200 (-1382 to 39) |  |
| Madhi, 2004 a | PCV9 (≥1) | <48 m, HIV uninfected | Pneumonia | 29 (-61 to 68) |  |
| **Coronavirus (any)** | | | | |  |
| Nunes, 2021 a | PCV9 (≥1) | ≤23 m, HIV infected | Pneumonia | 14 (-38 to 46) |  |
| Nunes, 2021 a | PCV9 (≥1) | ≤23 m, HIV uninfected | Pneumonia | 64 (23 to 83) |  |
| **Influenza** | | | | |  |
| - Pandemic H1N1 2009 influenza | | | | |  |
| Dominguez, 2013 b | PCV7/10/13 (≥1) | 6-60 m, at risk c | RTI | 36 (-379 to 92) |  |
| Dominguez, 2013 b | PCV7/10/13 (≥1) | 6-60 m, no risk | RTI | 58 (6 to 82) |  |
| Dominguez, 2013 b | PCV7/10/13 (fully vaccinated) | 6-60 m, at risk c | RTI | -10 (-993 to 89) |  |
| Dominguez, 2013 b | PCV7/10/13 (fully vaccinated) | 6-60 m, no risk | RTI | 53 (-2 to 81) |  |
| - Seasonal influenza | | | | |  |
| Madhi, 2004 a | PCV9 (≥1) | <48 m, HIV infected | Pneumonia | 42 (-8 to 69) |  |
| Madhi, 2004 a | PCV9 (≥1) | <48 m, HIV uninfected | Pneumonia | 39 (0 to 63) |  |
| Dominguez, 2013 b | PCV7/10/13 (≥1) | 6-60 m, at risk c | RTI | 15 (-1724 to 96) |  |
| Dominguez, 2013 b | PCV7/10/13 (any dose) | 6-60 m, no risk | RTI | -148 (-593 to 11) |  |
| Dominguez, 2013 b | PCV7/10/13 (fully vaccinated) | 6-60 m, at risk c | RTI | 9 (-2691 to 95) |  |
| Dominguez, 2013 b | PCV7/10/13 (fully vaccinated) | 6-60 m, no risk | RTI | -93 (-386 to 23) |  |
| **Metapneumovirus** | | | | |  |
| Madhi, 2006 a | PCV9 (≥1) | <57 m, HIV infected | LRTI | 47 (1 to 72) |  |
| Madhi, 2006 a | PCV9 (≥1) | <57 m, HIV uninfected | LRTI | 38 (14 to 56) |  |
| Madhi, 2006 a | PCV9 (≥1) | <57 m, HIV infected | Clinical pneumonia | 56 (12 to 78) |  |
| Madhi, 2006 a | PCV9 (≥1) | <57 m, HIV uninfected | Clinical pneumonia | 48 (7 to 67) |  |
| Madhi, 2006 a | PCV9 (≥1) | <57 m, HIV infected | Radiological pneumonia | 84 (26 to 96) |  |
| Madhi, 2006 a | PCV9 (≥1) | <57 m, HIV uninfected | Radiological pneumonia | 43 (-16 to 72) |  |
| Madhi, 2006 a | PCV9 (≥1) | <57 m, HIV infected | Severe pneumonia | 45 (-6 to 71) |  |
| Madhi, 2006 a | PCV9 (≥1) | <57 m, HIV uninfected | Severe pneumonia | 35 (5 to 56) |  |
| Madhi, 2006 a | PCV9 (≥1) | <57 m, HIV infected | Bronchiolitis | -24 (-83 to 237) |  |
| Madhi, 2006 a | PCV9 (≥1) | <57 m, HIV uninfected | Bronchiolitis | 25 (-20 to 53) |  |
| Madhi, 2006 a | PCV9 (≥1) | ≤6 m, HIV infected | LRTI | 34 (-295 to 89) |  |
| Madhi, 2006 a | PCV9 (≥1) | ≤6 m, HIV uninfected | LRTI | 6 (-83 to 51) |  |
| **Reference** | **Vaccine exposure (n. doses)** | **Population** | **Clinical outcome (Treatment setting)** | **VE value** |  |
| **Metapneumovirus** | | | | |  |
| Madhi, 2006 a | PCV9 (≥1) | 6-<12 m, HIV infected | LRTI | 43 (-93 to 83) |  |
| Madhi, 2006 a | PCV9 (≥1) | 6-<12 m, HIV uninfected | LRTI | 52 (7 to 75) |  |
| Madhi, 2006 a | PCV9 (≥1) | 1- <24 m, HIV infected | LRTI | 58 (-64 to 89) |  |
| Madhi, 2006 a | PCV9 (≥1) | 12 - <24 m, HIV uninfected | LRTI | 33 (-17 to 62) |  |
| Madhi, 2006 a | PCV9 (≥1) | >24 m, HIV infected | LRTI | 42 (-46 to 77) |  |
| Madhi, 2006 a | PCV9 (≥1) | >24 m, HIV uninfected | LRTI | 47 (-13 to 76) |  |
| **Parainfluenza (1-3)** | | | | |  |
| Madhi, 2004 a | PCV9 (≥1) | <48 m, HIV infected | Pneumonia | 41 (-17 to 70) |  |
| Madhi, 2004 a | PCV9 (≥1) | <48 m, HIV uninfected | Pneumonia | 44 (0 to 68) |  |
| **RSV** | | | | |  |
| Madhi, 2004 a | PCV9 (≥1) | <48 m, HIV infected | Pneumonia | 10 (-40 to 42) |  |
| Madhi, 2004 a | PCV9 (≥1) | <48 m, HIV uninfected | Pneumonia | 12 (-10 to 30) |  |

a. Efficacy, randomized controlled trial study; b. Effectiveness, case-control study; c. Risk conditions: solid organ or hematological neoplasia, chronic renal failure, transplantation, anemia, immunosuppressive therapy (chemotherapy or other treatment), HIV infection, diabetes, chronic heart disease and chronic lung disease, including asthma; HIV: human immunodeficiency virus; LRTI: lower respiratory tract infection; m: months; NR: not reported; PCV: pneumococcal conjugate vaccine; RSV: respiratory syncytial virus; RTI: respiratory tract infection; VE: vaccine effectiveness. **◦** In the case of the manuscripts Madhi 2004 and Madhi 2006, the age was not reported, so it is presumed from the information given in the paper.

## **Supplementary Table 8. Overall effect of PCVs on viral RTI in children (inpatient setting).**

| **Reference** | **Country** | **PCV (n. doses)** | **Population** | **Vaccine uptake** |  | **Incidence (per 100000)** | | | |  | **Adjusted by** |
| --- | --- | --- | --- | --- | --- | --- | --- | --- | --- | --- | --- |
|  |  |  |  |  | **RTI subtype** | **Pre-PCV** | | **Post-PCV** | | **IRR (95% CI)** |  |
|  |  |  |  |  |  | **Period** | **Value** | **Period** | **Value** |  |  |
| **Any virus** |  |  |  |  |  |  |  |  |  |  |  |
| Binks, 2020 | Australia | PCV10 vs PCV7(3+1) | ≤12 m | 3 doses: 90% in 2012, 4-doses (30 m of age): 80% in 2008, and 88% in 2017. | Pneumonia | 2006-2010▪a | 2.3 | 2010-2012▪ | 4.0 | 1.71 (1.32 to 2.20) | NR |
| Binks, 2020 | Australia | PCV13 vs PCV10 (3+1) | ≤12 m |  | Pneumonia | 2010-2012▪b | 4.0 | 2012-2015▪ | 2.8 | 0.70 (0.55 to 0.90) | NR |
| Binks, 2020 | Australia | PCV13 vs PCV7 (3+1) | ≤12 m |  | Pneumonia | 2006-2010▪c | 2.3 | 2012-2015▪ | 2.8 | 1.19 (0.94 to 1.52) | NR |
| Sigurdsson, 2020 | Iceland | PCV10 vs pre-PCV (3) | ≤36 m | >97% | Pneumonia | 2005-2010 | 80.0 | 2011-2015 | 84.0 | 1.05 (0.71 to 1.53) | NR |
| **Adenovirus** |  |  |  |  |  |  |  |  |  |  |  |
| Fathima, 2018 | Australia | PCV7/PCV13 vs pre-PCV (3) | ≤5 m, Aboriginal | NR | Pneumonia | 2000-2004 | 45.7 | 2005-2012 | 179.9 | 3.9 (0.9 to 17.3) | NR |
|  |  |  | 6-11 m, Aboriginal |  |  |  | 91.5 |  | 259.8 | 2.8 (0.9 to 8.3) |  |
|  |  |  | 12-23 m, Aboriginal |  |  |  | 57.3 |  | 59.1 | 1.0 (0.4 to 3.1) |  |
|  |  |  | 24-48 m, Aboriginal |  |  |  | 8.1 |  | 13.8 | 1.7 (0.30 to 8.2) |  |
|  |  |  | ≤192m, Aboriginal |  |  |  | 23.6 |  | 28.7 | 1.22 ¥ |  |
|  |  |  | ≤5 m, non-Aboriginal | NR | Pneumonia | 2000-2004 | 0.0 | 2005-2012 | 3.6 | 0.0 | NR |
|  |  |  | 6-11 m, non-Aboriginal |  |  |  | 17.3 |  | 16.3 | 0.9 (0.4 to 2.0) |  |
|  |  |  | 12-23 m, non-Aboriginal |  |  |  | 13.8 |  | 7.4 | 0.5 (0.3 to 1.1) |  |
|  |  |  | 24-48 m, non-Aboriginal |  |  |  | 2.6 |  | 2.3 | 0.9 (0.4 to 2.0) |  |
|  |  |  | ≤192m, non-Aboriginal |  |  |  | 4.7 |  | 2.2 | 0.47 ¥ |  |
| **Influenza** |  |  |  |  |  |  |  |  |  |  |  |
| Sigurdsson, 2020 | Iceland | PCV10 vs pre-PCV (3) | ≤36 m | >97% | Pneumonia | 2005-2010 | 6.0 | 2011-2015 | 2.0 | 0.30 (0.01 to 2.65) |  |
| Do, 2022 | Mongolia | PCV13 vs Pre-PCV (NR) | ≤12 m | NR | Pneumonia | 2015-2020 |  | 2015-2020 |  | 0.87 (0.52 to 1.46) | PCV13 introduction, district and time |
| **- Influenza A** |  |  |  |  |  |  |  |  |  |  |  |
| Fathima, 2018 | Australia | PCV7/PCV13 vs pre-PCV (3) | ≤5 m, Aboriginal | NR | Pneumonia | 2000-2004 | 68.5 | 2005-2012 | 25.7 | 0.4 (0.1 to 2.3) | NR |
|  |  |  | 6-11 m, Aboriginal |  |  |  | 205.8 |  | 129.9 | 0.6 (0.3 to 1.6) |  |
|  |  |  | 12-23 m, Aboriginal |  |  |  | 103.1 |  | 78.8 | 0.8 (0.3 to 1.8) |  |
|  |  |  | 24-48 m, Aboriginal |  |  |  | 36.6 |  | 24.7 | 0.7 (0.3 to 1.6) |  |
|  |  |  | ≤192m, Aboriginal |  |  |  | 56.2 |  | 25.4 | 0.45 ¥ |  |
|  |  |  | ≤5 m, non-Aboriginal | NR | Pneumonia | 2000-2004 | 8.7 | 2005-2012 | 1.8 | 0.2 (0.1 to 1.1) | NR |
|  |  |  | 6-11 m, non-Aboriginal |  |  |  | 12.1 |  | 5.4 | 0.5 (0.2 to 1.3) |  |
|  |  |  | 12-23 m, non-Aboriginal |  |  |  | 17.2 |  | 7.4 | 0.4 (0.2 to 0.8) |  |
|  |  |  | 24-48 m, non-Aboriginal |  |  |  | 8.5 |  | 4.6 | 0.5 (0.3 to 0.8) |  |
|  |  |  | ≤192m, non-Aboriginal |  |  |  | 8.9 |  | 3.2 | 0.36 ¥ |  |
| **- Influenza B** |  |  |  |  |  |  |  |  |  |  |  |
| Fathima, 2018 | Australia | PCV7/PCV13 vs pre-PCV (3) | ≤5 m, Aboriginal | NR | Pneumonia | 2000-2004 | 0.0 | 2005-2012 | 38.6 | … | NR |
|  |  |  | 6-11 m, Aboriginal |  |  |  | 57.2 |  | 38.9 | 0.7 (0.1 to 4.1) |  |
|  |  |  | 12-23 m, Aboriginal |  |  |  | 14.3 |  | 19.7 | 1.4 (0.1 to 13.2) |  |
|  |  |  | 24-48 m, Aboriginal |  |  |  | 0.0 |  | 8.9 | … |  |
|  |  |  | ≤192m, Aboriginal |  |  |  | 6.3 |  | 11.4 | 1.81 ¥ |  |
|  |  |  | ≤5 m, non-Aboriginal | NR | Pneumonia | 2000-2004 | 0.0 | 2005-2012 | 0.0 | 0.0 | NR |
|  |  |  | 6-11 m, non-Aboriginal |  |  |  | 0.0 |  | 3.6 | … |  |
|  |  |  | 12-23 m, non-Aboriginal |  |  |  | 2.2 |  | 1.4 | 0.6 (0.1 to 3.8) |  |
|  |  |  | 24-48 m, non-Aboriginal |  |  |  | 0.7 |  | 1.5 | 2.1 (0.5 to 9.7) |  |
|  |  |  | ≤192m, non-Aboriginal |  |  |  | 0.6 |  | 1.0 | 1.67 ¥ |  |
| **Metapneumovirus** |  |  |  |  |  |  |  |  |  |  |  |
| Fathima, 2018 | Australia | PCV7/PCV13 vs pre-PCV (3) | ≤5 m, Aboriginal | NR | Pneumonia | 2000-2004 | 57.8 | 2005-2012 | 77.1 | 1.3 (0.2 to 11.1) | NR |
|  |  |  | 6-11 m, Aboriginal |  |  |  | 0.0 |  | 64.9 | … |  |
|  |  |  | 12-23 m, Aboriginal |  |  |  | 0.0 |  | 59.1 | … |  |
|  |  |  | 24-48 m, Aboriginal |  |  |  | 28.7 |  | 11.2 | 0.4 (0.1 to 1.6) |  |
|  |  |  | ≤192m, Aboriginal |  |  |  | 14.7 |  | 15.7 | 1.07 ¥ |  |
|  |  |  | ≤5 m, non-Aboriginal | NR | Pneumonia | 2000-2004 | 25.9 | 2005-2012 | 9.8 | 0.4 (0.1 to 1.0) | NR |
|  |  |  | 6-11 m, non-Aboriginal |  |  |  | 21.9 |  | 19.0 | 0.9 (0.3 to 2.3) |  |
|  |  |  | 12-23 m, non-Aboriginal |  |  |  | 2.2 |  | 19.5 | 8.9 (1.2 to 64.5) |  |
|  |  |  | 24-48 m, non-Aboriginal |  |  |  | 6.4 |  | 8.1 | 1.3 (0.6 to 2.6) |  |
|  |  |  | ≤192m, non-Aboriginal |  |  |  | 5.9 |  | 5.4 | 0.92 ¥ |  |

| **Reference** | **Country** | **PCV** | **Population** | **Vaccine uptake** | **Incidence (per 100000)** | | | | | | **Adjusted by** |
| --- | --- | --- | --- | --- | --- | --- | --- | --- | --- | --- | --- |
|  |  |  |  |  | **RTI subtype** | **Pre-PCV** | | **Post-PCV** | | **IRR (95% CI)** |  |
|  |  |  |  |  |  | **Period** | **Value** | **Period** | **Value** |  |  |
| **Parainfluenza (1-4)** | | | | | | | | | | | |
| Fathima, 2018 | Australia | PCV7/PCV13 vs pre-PCV (3) | ≤5 m, Aboriginal | NR | Pneumonia | 2000-2004 | 137.0 | 2005-2012 | 90.0 | 0.7 (0.2 to 1.9) | NR |
|  |  |  | 6-11 m, Aboriginal |  |  |  | 182.9 |  | 116.9 | 0.6 (0.3 to 1.7) |  |
|  |  |  | 12-23 m, Aboriginal |  |  |  | 80.2 |  | 45.9 | 0.6 (0.2 to 1.6) |  |
|  |  |  | 24-48 m, Aboriginal |  |  |  | 8.1 |  | 8.9 | 1.1 (0.2 to 6.0) |  |
|  |  |  | ≤192m, Aboriginal |  |  |  | 45.3 |  | 15.7 | 0.35 ¥ |  |
|  |  |  | ≤5 m, non-Aboriginal | NR | Pneumonia | 2000-2004 | 13.9 | 2005-2012 | 4.5 | 0.3 (0.1 to 0.9) | NR |
|  |  |  | 6-11 m, non-Aboriginal |  |  |  | 20.8 |  | 10.9 | 0.5 (0.2 to 1.2) |  |
|  |  |  | 12-23 m, non-Aboriginal |  |  |  | 16.4 |  | 16.3 | 1 (0.6 to 1.7) |  |
|  |  |  | 24-48 m, non-Aboriginal |  |  |  | 7.3 |  | 6.4 | 0.9 (0.5 to 1.5) |  |
|  |  |  | ≤192m, non-Aboriginal |  |  |  | 8.9 |  | 4.7 | 0.53 ¥ |  |
| **Picornavirus** |  |  |  |  |  |  |  |  |  |  |  |
| Fathima, 2018 | Australia | PCV7/PCV13 vs pre-PCV (3) | ≤5 m, Aboriginal | NR | Pneumonia | 2000-2004 | 182.7 | 2005-2012 | 295.6 | 1.6 (0.7 to 3.6) | NR |
|  |  |  | 6-11 m, Aboriginal |  |  |  | 114.4 |  | 77.9 | 0.7 (0.2 to 2.2) |  |
|  |  |  | 12-23 m, Aboriginal |  |  |  | 22.9 |  | 52.5 | 2.3 (0.5 to 10.8) |  |
|  |  |  | 24-48 m, Aboriginal |  |  |  | 16.3 |  | 17.9 | 1.1 (0.3 to 3.7) |  |
|  |  |  | ≤192m, Aboriginal |  |  |  | 34.5 |  | 25.4 | 0.74 ¥ |  |
|  |  |  | ≤5 m, non-Aboriginal | NR | Pneumonia | 2000-2004 | 10.4 | 2005-2012 | 12.5 | 1.2 (0.5 to 3.1) | NR |
|  |  |  | 6-11 m, non-Aboriginal |  |  |  | 12.1 |  | 8.2 | 0.7 (0.3 to 1.9) |  |
|  |  |  | 12-23 m, non-Aboriginal |  |  |  | 10.4 |  | 12.5 | 1.2 (0.6 to 2.4) |  |
|  |  |  | 24-48 m, non-Aboriginal |  |  |  | 2.6 |  | 2.9 | 1.1 (0.5 to 2.5) |  |
|  |  |  | ≤192m, non-Aboriginal |  |  |  | 4.6 |  | 3.7 | 0.80 ¥ |  |
| **RSV** | | | | | | | | | | | |
| Fathima, 2018 | Australia | PCV7/PCV13 vs pre-PCV (3) | ≤5 m, Aboriginal | NR | Pneumonia | 2000-2004 | 479.5 | 2005-2012 | 488.3 | 1 (0.6 to 1.7) | NR |
|  |  |  | 6-11 m, Aboriginal |  |  |  | 480.3 |  | 363.8 | 0.8 (0.4 to 1.3) |  |
|  |  |  | 12-23 m, Aboriginal |  |  |  | 206.2 |  | 216.7 | 1.1 (0.6 to 1.9) |  |
|  |  |  | 24-48 m, Aboriginal |  |  |  | 81.4 |  | 69.6 | 0.9 (0.5 to 1.5) |  |
|  |  |  | ≤192m, Aboriginal |  |  |  | 145.1 |  | 73.1 | 0.50 ¥ |  |
|  |  |  | ≤5 m, non-Aboriginal | NR | Pneumonia | 2000-2004 | 121.4 | 2005-2012 | 80.1 | 0.7 (0.5 to 0.9) | NR |
|  |  |  | 6-11 m, non-Aboriginal |  |  |  | 91.8 |  | 60.7 | 0.7 (0.5 to 0.9) |  |
|  |  |  | 12-23 m, non-Aboriginal |  |  |  | 79.4 |  | 84.9 | 1.1 (0.8 to 1.4) |  |
|  |  |  | 24-48 m, non-Aboriginal |  |  |  | 28.8 |  | 30.9 | 1.1 (0.8 to 1.4) |  |
|  |  |  | ≤192m, non-Aboriginal |  |  |  | 42.6 |  | 21.9 | 0.51 ¥ |  |
| Binks, 2020 | Australia | PCV10 vs PCV7(3+1) | ≤12 m | 3 doses: 90% in 2012, 4-doses (30 m of age): 80% in 2008, and 88% in 2017. | Pneumonia | 2006-2010▪a | 4.9 | 2010-2012▪ | 4.9 | 1.07 (0.80 to 1.44) | Adjusted for secular trends |
| Binks, 2020 | Australia | PCV13 vs PCV10 (3+1) | ≤12 m |  | Pneumonia | 2010-2012▪b | 4.9 | 2012-2015▪ | 4.9 | 1.08 (0.79 to 1.45) |  |
| Binks, 2020 | Australia | PCV13 vs PCV7 (3+1) | ≤12 m |  | Pneumonia | 2006-2010▪c | 4.9 | 2012-2015▪ | 4.9 | 1.15 (0.72 to 1.85) |  |
| Do, 2022 | Mongolia | PCV13 vs Pre-PCV (NR) | ≤12 m | NR | Pneumonia | 2015-2020 |  | 2015-2020 |  | 0.90 (0.64 to 1.25) | PCV13 introduction, district and time |
| Weinberger, 2015 | US | PCV7 vs Pre-PCV (NR) | ≤11 m | NR | RSV- coded LRTI | 1997-2000 |  | 2004-2009 |  | 0.82 (0.77 to 0.87) | NR |
| Weinberger, 2015 | US |  | 3-11 m |  | RSV- coded LRTI | 1997-2000 |  | 2004-2009 |  | 0.92 (0.85 to 0.97) | NR |
| Weinberger, 2015 | US |  | 12-23 m |  | RSV- coded LRTI | 1997-2000 |  | 2004-2009 |  | 0.82 (0.75 to 0.89) | NR |
| Foote, 2015 | US | PCV7 vs Pre-PCV (NR) | ≤48 m, American Indian and Alaska Native | 4 doses: 71% in 2009 and 75% in 2011. | RSV-coded LRTI | 1998-1999 |  | 2009-2010 |  | 0.64** | NR |
| Foote, 2015 | US |  | ≤48 m, US infants* | 4 doses: 83% in 2009 and 85% in 2011. | RSV-coded LRTI | 1998-1999 |  | 2009-2010 |  | 0.80 | NR |

a: pre-PCV10 is PCV7 period; b: pre-PCV13 is PCV10 period; c: pre-PCV13 is PCV7 period; IRR: incidence rate ratio; LRTI: lower respiratory tract infection; m: months. NR: not reported; PCV: pneumococcal conjugate vaccine; RSV: respiratory syncytial virus; RTI: respiratory tract infection; US: United States of America; *US non-Hispanic white child population; ** p<0.01, ▪per 100 py; ¥: IRR calculated from incidence rates given by the article; ◊: IRR calculated from vaccine impact provided by the article.
